# Study on β-Cyclodextrin-Functionalized Molten Salt Nitrogen-Doped Biochar and Its Adsorption Performance and Mechanism

**DOI:** 10.3390/molecules31081284

**Published:** 2026-04-14

**Authors:** Sining Li, Yong Huang, Qiushuang Cui, Ke Jin, Hanyu Wei, Wen Liu, Huan Li, Ruyun Bai

**Affiliations:** 1 State Key Laboratory of Chemistry and Utilization of Carbon Based Energy Resources, College of Chemistry, Xinjiang University, Urumqi 830017, China; lsn970525@163.com (S.L.);; 2 College of Civil Engineering and Architecture, Xinjiang University, Urumqi 830017, China; 3 Fangda Special Steel Technology Co., Ltd., Nanchang 330012, China; 4 Henan Province Water Conservancy Second Engineering Bureau Group Co., Ltd., Zhengzhou 450016, China

**Keywords:** β-CD functionalization, tetracycline, methylene blue, adsorption mechanism

## Abstract

In this study, we prepare N–doped biochar loaded with β-CD, using cotton stalks as a carbon source, and evaluate its removal efficiency for tetracycline (TC) and methylene blue (MB) from aqueous solutions. This composite uniquely integrates molten salt activation, nitrogen doping, and β-CD grafting, resulting in an exceptionally high specific surface area of 1943 m^2^/g and abundant active sites. The findings reveal that β-CD-NKBC-1.5 (5 g of N–doped biochar loaded with 1.5 g of β-CD) demonstrates remarkable capabilities for both TC and MB removal across an extensive pH spectrum, reaching peak adsorption levels of 1269.8 and 969.4 mg/g at 308.15 K, respectively—outperforming most previously reported biochar-based adsorbents. The adsorption process is well described by the pseudo-second-order and Langmuir models, indicating that monolayer chemisorption is the dominant mechanism. β-CD-NKBC-1.5 exhibits preferential adsorption for TC and MB and maintains high adsorption efficiency even with coexisting ions (Na^+^, K^+^, Ca^2+^, Mg^2+^, and SO_4_^2−^) at concentrations up to 500 mg/L. The adsorption mechanism involves Lewis acid–base interactions, hydrogen bonding, π–π stacking, and pore filling.

## 1. Introduction

Tetracyclines (TCs) are widely used in medical treatment due to their potent antibacterial effects and low cost [1]. However, their slow degradation, complex metabolic pathways, and limited microbial degradation lead to environmental persistence, contributing to bacterial resistance and posing risks to ecosystems and human health [2]. Untreated textile and dyeing wastewater contains high amounts of dyes that are detrimental to human health, aquatic species, and ecosystems. Methylene blue (MB) is widely used in printing, dyeing, textile, and bio-dyeing processes [3], making its removal from the environment essential [4]. Consequently, the pollution caused by antibiotics and dyes entering water bodies has become a global issue, and the removal of these contaminants is crucial for safeguarding the environment and human health [5]. These two pollutants are frequently co-detected in industrial and agricultural wastewater. Their structural differences—with TC containing multiple ionizable functional groups and MB possessing a planar aromatic structure—enable a comprehensive evaluation of the adsorption mechanisms of β-CD-NKBC composites [6]. Understanding the adsorption behaviors for both TC and MB is essential for developing versatile and efficient biochar-based adsorbents suitable for real-world wastewater treatment applications containing diverse organic contaminants [7]. The development of ecologically acceptable and cost-effective pollution removal strategies is critical [8], and numerous approaches have been used, including adsorption [9,10], biodegradation [11], photocatalysis [12], electrocatalysis [13], and advanced oxidation processes [14]. Biochar, a renewable carbon-based adsorbent material, has demonstrated excellent effectiveness in wastewater pollutant adsorption and removal [15]. Biochar’s porous structure and functional groups enhance its adsorption capacity for organic contaminants, making it a promising solution for wastewater treatment [16].

Recent research has shown that biochar may remove trichloromethane and methyl bromide, offering a potential solution to these challenges [17]. When it comes to tetracycline adsorption, Liu and colleagues’ grape-derived biochar adsorbed 52.90 mg/g of the compound [18], while Li et al.’s bamboo-based counterpart proved far more effective, adsorbing a substantial 310.70 mg/g of the compound [19]. The presence of functional groups featuring heteroatoms such as nitrogen, oxygen, and sulfur on the biochar surface enables the formation of complexes with contaminants, which in turn enhances the elimination of contaminants from the environment. Furthermore, nitrogen doping also helps to enhance the adsorption capacity of biochar, as nitrogen species can introduce additional defects and active sites to anchor TC molecules; moreover, different types of nitrogen sources, aided by activators, can play a dual role in promoting the formation of a porous structure [20]. Zhang et al. [21] synthesized magnetic nitrogen-doped biochar (MNSB) via a one-pot pyrolysis process using sugarcane bagasse and red mud as feedstocks. Adsorption kinetics and isotherm analyses indicated that the adsorption process was primarily governed by chemical adsorption, with MNSB exhibiting a theoretical maximum adsorption capacity of 143.9 mg/g for TC. Xiong et al. successfully synthesized magnetic iron-loaded BC (MBC700) for the adsorption of butylcarbamate. The results demonstrated that MBC700 rapidly removed butylcarbamate within 30 min, achieving a removal capacity of up to 158.5 mg/g [22]. Grafting modification involves attaching specific functional molecules or polymers onto the biochar surface. This introduces new functional groups and optimizes the surface and pore structure, thereby enhancing adsorption efficiency and selectivity, broadening the application scope, and improving environmental adaptability [23]. β-Cyclodextrins (β-CDs) are ring-shaped carbohydrate molecules connected through α-1,4 glycosidic linkages, featuring water-repelling interiors and water-attracting exteriors [24]. These compounds have gained popularity in pollution remediation due to their low cost, biodegradability, and environmental friendliness [25]. However, the high water solubility of β-CD makes its recovery difficult; therefore, researchers often employ cross-linking or immobilization techniques to overcome this issue [26]. Rohith et al. [27] developed an advanced adsorbent by combining β-CD with lotus agricultural waste, demonstrating high efficiency and durability in removing hazardous dyes. Chen et al. [28] synthesized β-CD@MBCP particles using EDTA as a cross-linker. These particles exhibited maximum adsorption capacities of 547.4 mg/g for Pb(II) and 859.6 mg/g for MB, demonstrating excellent performance for the removal of these contaminants. Benedicto et al. [29] used two methods to functionalize coffee grounds with β-cyclodextrin. Compared to the non-functionalized material, the functionalized samples (BCG(OH) and BCE(OH)) showed enhanced adsorption capacity for cetylpyridinium chloride (CPC) but lower adsorption for sodium dodecylbenzenesulfonate. The adsorption capacity of BCE(OH) proved to be superior, hitting maximum adsorption levels of 113.6 mg/g and 187.1 mg/g, respectively. Additionally, the findings clearly demonstrated that epichlorohydrin was the more effective cross-linking agent for β-CD. Wu et al. [30] used β-cyclodextrin-functionalized biochar (β-BC) to treat wastewater co-contaminated with heavy metals and methyl orange (MO), achieving effective removal of 20 mg/L for heavy metals and 150 mg/L for MO in a continuous adsorption process. The addition of β-BC reduced the residual heavy metal concentration to 0.05 mg/L, compared to 0.16 mg/L in the control group. Furthermore, the MO removal rate increased from 87.92–94.11% to 96.79–98.84% with the addition of β-BC. Cotton stalk is composed primarily of cellulose, hemicellulose, and lignin [31]. Typically, cotton stalk contains ∼32–46% cellulose, 20–28% hemicellulose, and 15–30% lignin [32]. Mary et al. [33] investigated the capacity of cotton stalks to remove and eliminate copper from agricultural and livestock wastewater. They determined that the optimal adsorption conditions were a solution pH of 5.5, an adsorbent dosage of 0.6 g, and an adsorption time of 60 min. Under these conditions, a removal rate of approximately 66.5% was achieved in synthetic samples with an initial copper concentration of 50 mg/L. Zhang et al. [34] prepared a selective adsorption material for dyeing wastewater by pre-treating cotton stalks with sodium chlorite and modifying them with ethyleneamine. This material exhibited a distinctive selective adsorption capacity for Congo red, reaching 997.1 mg/g, with electrostatic interactions, π–π bonds, and hydrogen bonds playing significant roles in the adsorption process. The Xinjiang region of northwestern China produces approximately 17 million tons of cotton stalks annually, providing abundant biomass for biochar production and reducing agricultural waste [35]. Despite the progress in biochar modification, the combination of molten salt activation, nitrogen doping, and β-CD functionalization has not been systematically explored for the removal of antibiotics and dyes.

In this study, β-CD-NKBC composites were prepared via molten salt activation, nitrogen doping, and surface functionalization with β-CD. The study then investigated adsorption performance and mechanisms for TC and MB. The adsorption process was comprehensively studied using kinetics, thermodynamics, and various characterization techniques. The results demonstrated the material’s excellent versatility and high adsorption capacity over a wide pH range and in complex ionic environments. This innovative approach synergistically combines the advantages of molten salt activation (creating well-developed porosity), nitrogen doping (introducing Lewis basic sites), and β-CD grafting. This material demonstrates exceptional contaminant removal capabilities even in complex water systems containing multiple pollutants simultaneously, paving the way for creating advanced, sustainable, and eco-friendly adsorbents that could revolutionize water purification technologies.

## 2. Results and Discussion

### 2.1. Characterization Analysis of β-CD-NKBC

**Figure 1:**
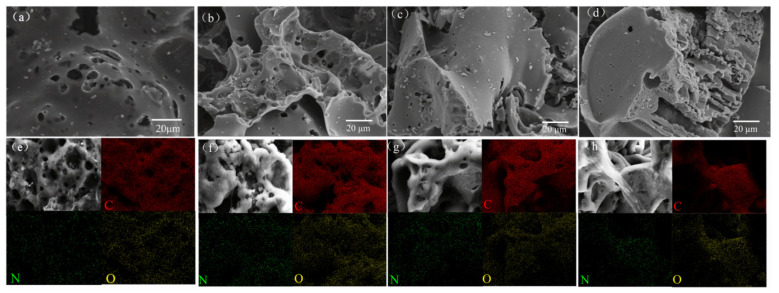
SEM of (**a**) NKBC; (**b**) β-CD-NKBC-2; (**c**) β-CD-NKBC-1.5; (**d**) β-CD-NKBC-1; Mapping of (**e**) NKBC; (**f**) β-CD-NKBC-2; (**g**) β-CD-NKBC-1.5; (**h**) β-CD-NKBC-1.

Figure 1 displays the SEM of β-CD-NKBC. The β-CD cross-linking reaction with ECH under alkaline conditions did not significantly change the overall morphology of the biochar compared to the pristine sample [10]. Figure 1a–d show scanning electron microscope (SEM) images of NKBC, which, like β-CD-NKBC, exhibits an amorphous, highly porous powdery morphology with particles of varying sizes and a rough surface. This characteristic allows for a greater contact area with the liquid phase, which improves the mass transfer efficiency. Figure 1e,f demonstrate that, when β-CD forms cross-linked structures with ECH, it coats the NKBC surface and significantly reduces the content of C, N, and O elements. The mapping in Figure 1f–h shows a rising trend in the content of C, N, and O elements. This indicates that mild β-CD functionalization changes the surface element distribution, leading to an increase in the number of chemical groups associated with these elements. Modifying NKBC with a large fraction of β-CD and ECH reduces C, N, and O levels further. Excessive β-CD-ECH cross-linking structures can form thick layers on the NKBC surface, lowering the surface content of C, N, and O elements. β-CD functionalization has little impact on the morphology of NKBC but drastically changes the surface composition of the C, N, and O components.

**Figure 2:**
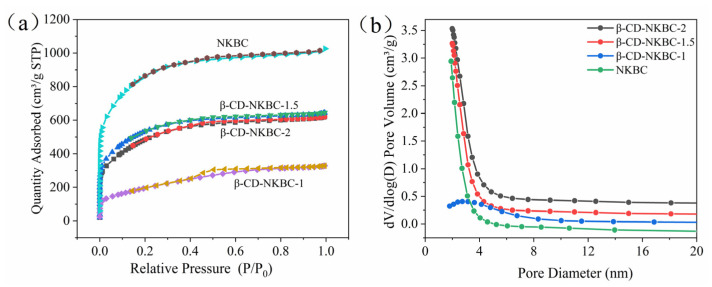
(**a**) N_2_ adsorption–desorption profiles; (**b**) pore size distribution of NKBC and β-CD-NKBC-2, 1.5, and 1.

The pore structure of the prepared β-CD-NKBC composites was systematically characterized using nitrogen adsorption–desorption isotherms, with the results shown in Figure 2. According to the IUPAC classification criteria, the isotherms of β-CD-NKBC-2 and β-CD-NKBC-1.5 exhibit typical Type I characteristics, indicating that micropores dominate their structure. In contrast, β-CD-NKBC-1 exhibited a composite isotherm of both Type I and Type IV, with a distinct hysteresis loop appearing at relative pressures above 0.4, suggesting the coexistence of micropores and mesopores in its structure. This result is consistent with its broader pore size distribution and lower micropore fraction (see Table 1). The pore size distribution curves further reveal structural differences between the samples across different pore size ranges. β-CD-NKBC-1.5 exhibits a high pore volume in both the micropore and mesopore ranges, with particularly significant pore development in the 0–5 nm range, providing abundant adsorption sites and excellent mass transfer pathways. In contrast, the pore size distribution of β-CD-NKBC-1 shifts towards larger pore sizes, indicating that excessive or insufficient β-CD modification may lead to the collapse or blockage of the pore structure.

Table 1 summarizes the specific surface area and pore structure parameters of each biochar sample. The specific surface area was calculated using the BET method, and the Barrett–Joyner–Halenda (BJH) model was employed to analyze the mesopore distribution. The results show that β-CD-NKBC-1.5 possesses the highest specific surface area (1943 m^2^/g) and micropore surface area (1825 m^2^/g), with a total pore volume and micropore volume of 0.997 cm^3^/g and 0.866 cm^3^/g, respectively. Compared with the unmodified NKBC, β-CD-NKBC-1.5 exhibited a slight decrease in the micropore fraction but a significant increase in the mesopore fraction, indicating that the successful incorporation of β-CD not only preserved the original micropore structure but also created new mesopore channels through the cross-linking reaction. It is worth noting that, although β-CD-NKBC-2 also retained a high specific surface area (1773 m^2^/g), its pore volume was slightly lower than that of β-CD-NKBC-1.5, suggesting that the introduction of an appropriate amount of β-CD helps to optimize the pore structure, whereas excessive modification may lead to the partial coverage or blockage of pores [4]. Conversely, the specific surface area and pore volume of β-CD-NKBC-1 decreased significantly (711 m^2^/g, 0.507 cm^2^/g), further confirming the regulatory effect of β-CD dosage on the pore structure.

**Figure 3:**
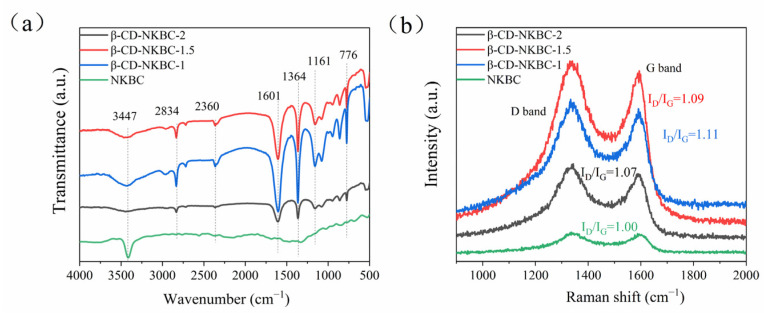
Spectra of NKBC, β-CD-NKBC-2, 1.5, and 1: (**a**) FTIR spectra; (**b**) Raman spectra.

Figure 3a shows that, compared with NKBC, the absorption peak at 3447 cm^−1^ is significantly weaker in β-CD-NKBC. This is due to the formation of ether bonds from the −OH groups of β-CD during cross-linking with ECH, which reduces the number of −OH groups and thus the intensity of the O–H stretching vibration at 3447 cm^−1^ [36]. The peak at 2834 cm^−1^, corresponding to aliphatic C–H symmetric stretching, shows no significant change in position or intensity after cross-linking, indicating the stability of these groups. The 1601 cm^−1^ absorption peak represents the aromatic ring’s C=C stretch. The peak at 1364 cm^−1^ signifies the symmetrical C–O stretch in the β-CD and ECH cross-linking arrangement, whereas the nearby peak at 1364 cm^−1^ corresponds to the C–O stretch resulting from the interaction of the ECH’s epoxy ring with the hydroxyl group of β-CD [37]. The peak at 776 cm^−1^ corresponds to aromatic C–H out-of-plane bending, confirming the presence of aromatic rings. The peak validates the existence of an aromatic ring in the substance. The aromatic ring in the β-CD molecule and the aromatic carbon skeleton in the biochar produce typical absorption at this wave number, confirming the material’s aromatic structure and stability during cross-linking [38].

Figure 3b clearly shows that the I_D_/I_G_ ratios in the Raman spectra of NKBC, β-CD-NKBC-2, 1.5, and 1 are 1.0, 1.09, 1.11, and 1.07, respectively. The β-CD molecular structure contains functional groups, including hydroxyl groups, which chemically bond to NKBC surface atoms or groups, increasing the number of lattice defects. Additionally, cross-linking polymers on the NKBC surface cause local structural disturbances [39], leading to an increase in the number of defects and a peak I_D_/I_G_ value of 1.11. As the amount of β-CD decreases, the cross-linked polymer produced between β-CD and ECH cannot fully cover the NKBC’s surface. The NKBC particles that are not coated by β-CD agglomerate, increasing their contact area. This facilitates the fusion of carbon structures to some extent, resulting in a more compact structure and reducing the number of surface defects. However, insufficient β-CD leads to incomplete cross-linking, limiting defect formation [40]. The uncoated NKBC portions, with higher graphitization, contribute to the lower I_D_/I_G_ ratio.

**Figure 4:**
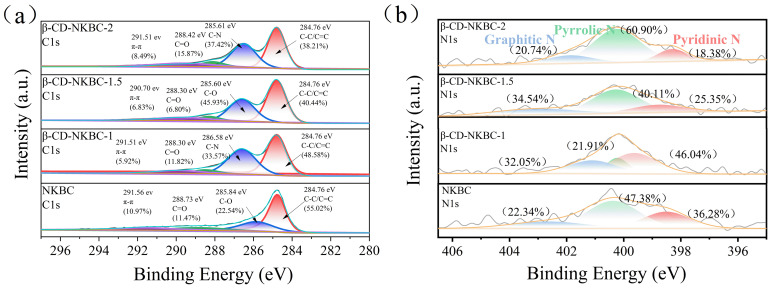
XPS spectra of NKBC, β-CD-NKBC-2, 1.5, and 1: (**a**) C1s, (**b**) N1s.

XPS analysis was performed to determine the chemical states and elemental composition of β-CD-NKBC, providing insights into its adsorption performance (Figure 4). The C1s spectrum shows four distinct peaks: at 284.8 eV (C–C), 285.6 eV (C–N), 288.4 eV (C=O), and 291.5 eV (π–π*). The N1s spectrum, on the other hand, consists of three peaks: graphite N, pyridinic N, and pyrrole N. From β-CD-NKBC-2 to β-CD-NKBC-1, as the proportion of β-CD gradually decreased, the percentage of the C-N peak first increased and then decreased, approaching the fitted peak percentage of NKBC. With decreasing β-CD content, the graphitic N content increased from 20.74% to 34.54%, and pyridinic N increased from 18.36% to 46.04% (for β-CD-NKBC-1). Reducing the quantity of β-CD resulted in a thin and discontinuous covering layer on the surface of NKBC, as well as an increase in uncovered graphite and pyridine. In contrast, the content of pyrrole N decreased sharply from 60.90% in β-CD-NKBC-2 to 21.91% in β-CD-NKBC-1. This was mainly due to the fact that a large number of cross-linked polymers generated during the cross-linking reaction between β-CD and ECH altered the surface of the NKBCs, which reduced the structural stability of the pyrrole N. Some of the pyrrole N was transformed to form a more stable N, such as graphitic N or pyridinic N. Moreover, with minimal β-CD and substantial NKBC, the surface configuration of NKBC active sites transformed. Most pyrrole N sites transformed into alternative N configurations, substantially reducing pyrrole N. The findings indicated uniform distribution of β-CD across the NKBC biochar’s surface, leading to the successful synthesis of β-CD-NKBC composites.

### 2.2. Adsorption Performance Analysis

**Figure 5:**
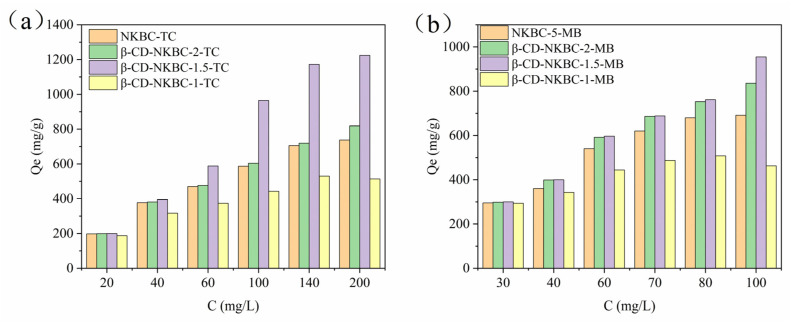
Adsorption capacities of NKBC, β-CD-NKBC-2, 1.5, and 1 for varying concentrations of (**a**) TC and (**b**) MB.

Figure 5 shows the equilibrium adsorption capacities of NKBC, β-CD-NKBC-2, 1.5, and 1 for TC as a function of the initial concentration. The capacities increased from 197.4, 198.7, 199.4, and 187.3 mg/g to 737.4, 817.9, 1224.3, and 513.5 mg/g, respectively. With increasing MB concentrations spanning from 30 to 100 mg/L, the equilibrium adsorption capacity saw a significant leap, climbing from 295.2, 298.5, 299.9, and 293.5 mg/g to 691.2, 835.8, 984.9, and 462.7 mg/g, respectively. The equilibrium adsorption capacities of β-CD-NKBC-2, 1.5, and 1 for TC and MB first increased and then decreased. This was due to the fact that, when the proportion of β-CD was small, β-CD and the ECH polymer masked the active sites introduced by N doping and KOH activation, reducing the adsorption performance [41]. When β-CD was added, the polymer formed by β-CD-NKBC-1.5 was uniformly distributed on the surface of the biochar. The cyclic cavity and cross-linked network of β-CD increased the mesopore volume, which led to an increase in the micropores or mesopores. The hydroxyl groups and ether bonds generated by the cross-linking of β-CD enhanced the surface polarity and provided more adsorption sites, resulting in an increase in the adsorption performance for TC and MB. Smaller β-CD caused charcoal agglomeration and reduced the number of active sites, resulting in decreased adsorption performance. β-CD-NKBC-1.5 showed superior adsorption performance for TC/MB. To further evaluate the application potential of β-CD-NKBC-1.5, its adsorption performance was systematically compared with that of various reported adsorbents (Table 2). The maximum adsorption capacities of β-CD-NKBC-1.5 for TC and MB reached 1269.8 mg/g and 969.4 mg/g, respectively. These values exceed those of most previously reported adsorbents, demonstrating β-CD-NKBC-1.5’s strong potential for simultaneous removal of TC and MB from contaminated water.

### 2.3. pH Impact on TC and MB Adsorption by β-CD-NKBC-1.5

**Figure 6:**
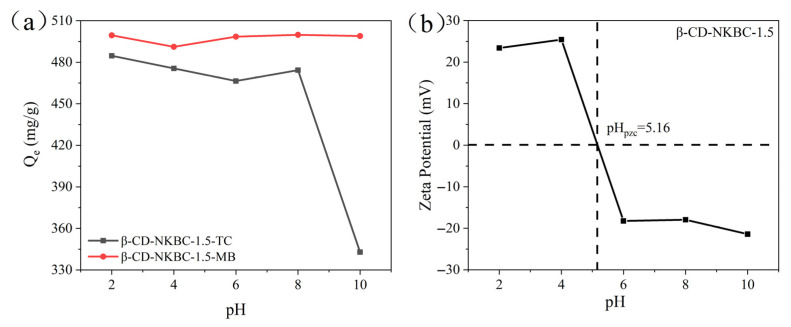
Adsorption performance of β-CD-NKBC-1.5 at different pH levels: (**a**) adsorption performance plot; (**b**) Zeta potential plot.

Figure 6 illustrates the Zeta potential and adsorption of β-CD-NKBC-1.5 on MB at various pH levels. The pH_PZC_ value for β-CD-NKBC-1.5 is 5.16. The adsorption capacity of TC exhibits significant dependence on the pH: it gradually increases as the pH rises from 2 to 7 during the initial stage, peaks at pH ≈ 7, and subsequently decreases with further pH elevation. When the solution pH falls below pH_PZC_, the β-CD-NKBC-1.5 surface carries a positive charge, leading to electrostatic repulsion with the positively charged TCH^3+^ species, thereby reducing the adsorption efficiency. Within the pH range of 5.16–7.7, the adsorbent surface carries a negative charge, while TC predominantly exists as an amphoteric ion; the strong electrostatic repulsion vanishes, and hydrogen bonding and π–π interactions jointly promote a high adsorption capacity [54]. When the pH exceeds 7.7, both the adsorbent surface and TC species carry negative charges, intensifying electrostatic repulsion and consequently diminishing the adsorption capacity [55]. For the cationic dye MB, its adsorption capacity remains relatively stable across the entire pH range studied, showing only a slight decrease at low pH values and a gradual increase at higher pH values. Below pH_PZC_, both β-CD-NKBC-1.5 (positively charged) and MB (cationic) experience electrostatic repulsion; nevertheless, the material exhibits a significant adsorption capacity, indicating that alternative mechanisms such as hydrogen bonding, π–π stacking, and host–guest encapsulation play crucial roles. When the pH rises above pH_PZC_, the adsorbent surface becomes negatively charged, enhancing electrostatic attraction with cationic MB molecules, thereby increasing the adsorption capacity. MB adsorption shows a relatively minor variation with the pH, indicating that β-CD-NKBC-1.5 exhibits stable MB adsorption performance across a broad pH range. This is attributed to synergistic effects from multiple interactions.

### 2.4. Adsorption Kinetics of β-CD-NKBC-1.5

**Figure 7:**
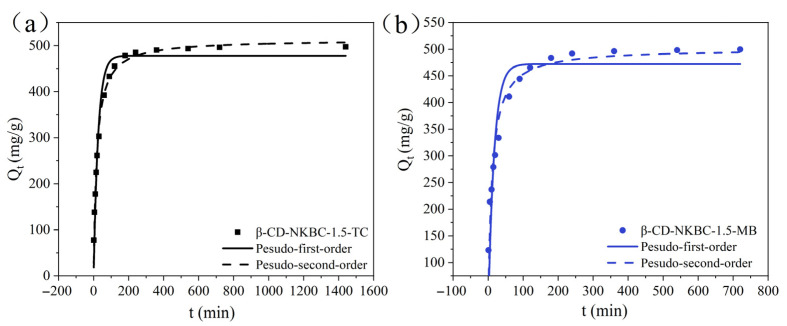
Kinetic model fitting curves of β-CD-NKBC-1.5: (**a**) TC; (**b**) MB.

Figure 7 shows the adsorption kinetics of TC and MB onto β-CD-NKBC-1.5. The adsorption capacity of β-CD-NKBC-1.5 on TC and MB gradually increased and stabilized over time. When adsorbed for 30 min, the adsorption capacity of TC and MB was 302.8, and 333.8 mg/g, respectively; when adsorbed for 180 min, the adsorption capacity of TC and MB was 478.2 and 483.6 mg/g, respectively; and after 720 min, the removal rate reached more than 98%, and adsorption equilibrium was achieved. The hydrophobic cavity of β-CD encapsulates the hydrophobic groups of TC, while hydroxyl and ether bonding improves material dispersibility, enhances contact with contaminants, and increases the adsorption rate. Table 3 reveals that the pseudo-second-order model provides a superior fit in depicting the adsorption processes of TC and MB onto β-CD-NKBC-1.5 as compared to the pseudo-first-order model, indicating that chemisorption is the rate-limiting step.

**Figure 8:**
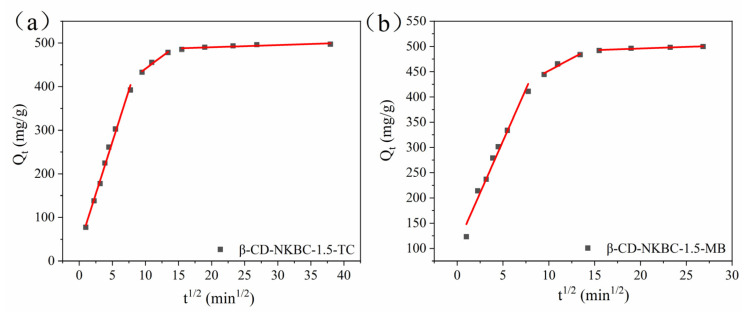
β-CD-NKBC-1.5 internal diffusion model fit: (**a**) TC; (**b**) MB.

Figure 8 displays the intra-particle diffusion model for β-CD-NKBC-1.5, while Table 4 lists the values of K_int_, C_i_, and R^2^. The adsorption process is divided into three phases: The slopes decrease in the order of the K_int1_ > K_int2_ > K_int3_ law, while the intercepts increase in the order of C_1_ < C_2_ < C_3_, indicating that intraparticle diffusion is the rate-limiting step [56]. The higher K_int_ value indicates a rapid diffusion of TC and MB to the outer surface of β-CD-NKBC-1.5. In the second stage, the slope decreases as TC and MB molecules diffuse from the external surface into the internal pore structure. This corresponds to internal particle diffusion. Finally, the smaller K_int3_ represents the final adsorption equilibrium phase, in which intra-particle diffusion slows to equilibrium.

### 2.5. Adsorption Thermodynamics of β-CD-NKBC-1.5

**Figure 9:**
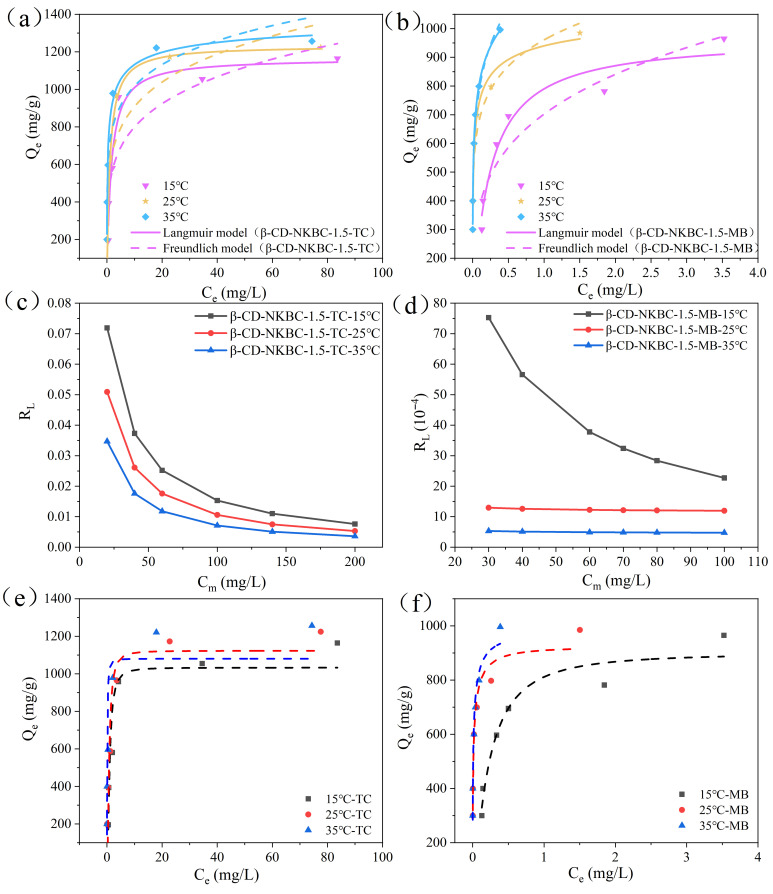
Langmuir and Freundlich adsorption isotherms for β-CD-NKBC-1.5 (**a**) TC and (**b**) MB adsorption patterns; plot of separation factor (RL) versus initial concentration for (**c**) TC and (**d**) MB; D-R model fitting (**e**) for TC and (**f**) for MB.

Three isotherm models were used to fit the adsorption data of β-CD-NKBC-1.5, as shown in Figure 9, and the fitted parameters are listed in Table 5. The Langmuir model of β-CD-NKBC-1.5 showed higher R^2^ values for TC (0.991–0.994) and MB (0.993–0.997) compared to the Freundlich model (0.807–0.911, 0.897–0.987), indicating monolayer adsorption. Figure 9c,d show that β-CD-NKBC-1.5 adsorbed well on both TC and MB, with an RL range of 0–1. Table 6 shows that β-CD-NKBC-1.5 has n_F_ values greater than 1 for both TC and MB, indicating physisorption. The adsorption 1/n_F_ values of β-CD-NKBC-1.5 at different temperatures were 0.153–0.208 for TC and 0.144–0.260 for MB, indicating a degree of heterogeneity.

Figure 9a,b show that the adsorption capacity of β-CD-NKBC-1.5 for TC and MB increased with the temperature. When the temperature was raised from 288.15 K (1165.7 mg/g) to 308.15 K (1269.8 mg/g), a Qmax rise of 104.1 mg/g was observed for TC, while the Qmax adsorption for MB increased from 915.2 mg/g to 969.4 mg/g. The D-R model’s adsorption energy (E) for TC ranged from 0 to 8 KJ/mol, indicating physical adsorption. For MB, E was greater than 8 KJ/mol and increased with the temperature, indicating ion exchange as the primary mode of adsorption.

**Figure 10:**
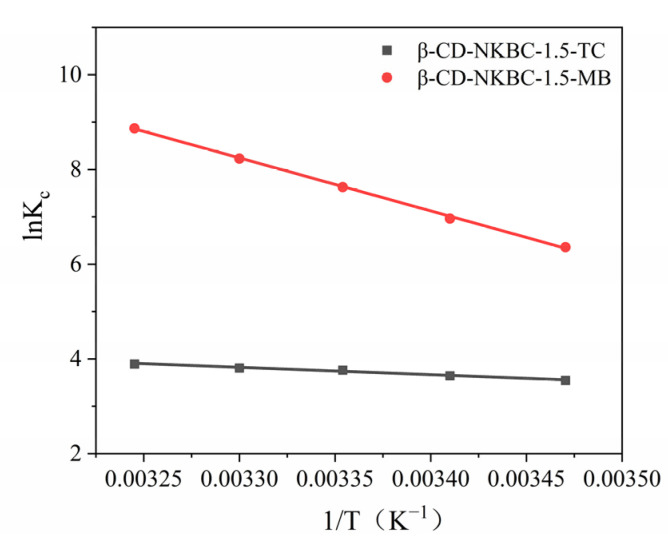
Plot of lnKc and 1/T for β-CD-NKBC-1.5.

Table 6 shows the relationship between the thermodynamic constants, lnKc and 1/T, following the adsorption of TC and MB, as shown in Figure 10. As the temperature climbs from 288.15 K to 308.15 K, the van’t Hoff equation reveals that ΔH^0^ > 0 (specifically 12.850 and 92.650 KJ/mol), which suggests that β-CD-NKBC-1.5’s attachment to TC and MB is an endothermic process. In addition, the adsorption mechanism is revealed to be ΔG^0^ < 0. The decrease in ΔG^0^ with increasing temperature suggests that higher temperatures favor adsorption. The thermodynamic parameter ΔS^0^ > 0 (74.171 and 374.152 J/(mol·K)) indicates a decrease in the degree of freedom in the adsorption process.

### 2.6. Effect of Coexisting Cation–Anion Pairs on the Adsorption Performance of β-CD-NKBC-1.5

**Figure 11:**
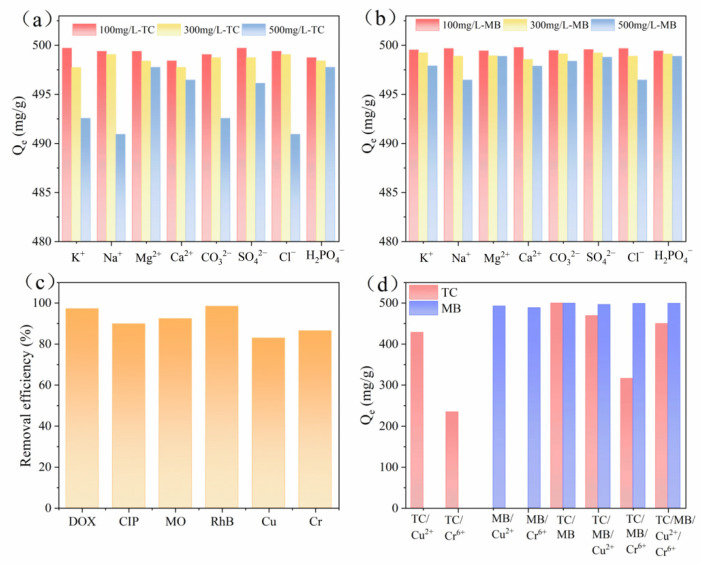
(**a**,**b**) Effect of coexisting anions on TC adsorption using β-CD-NKBC-1.5, (**c**) suitability of β-CD-NKBC-1.5 for multiple pollutants, (**d**) co-existing adsorption performance graphs for heavy metal ions.

Ionic strength can affect adsorption efficiency by altering ionization and surface charge, as well as adsorbent–adsorbate interactions [57]. This study examined how different inorganic ion concentrations (Na^+^, K^+^, Ca^2+^, Mg^2+^, SO_4_^2−^, H_2_PO_4_^−^, Cl^−^, and CO_3_^2−^) affected the performance of β-CD-NKBC-1.5 for TC/MB removal. As shown in Figure 11a,b, at a concentration of 100 mg/L, all competing ions had insignificant effects on TC and MB adsorption. At an ion concentration of 500 mg/L, the presence of Na^+^, K^+^, and Cl^−^ salts appeared to inhibit TC and MB adsorption, most likely owing to the ‘salting-out’ effect that rendered the contaminants less soluble in solution and consequently reduced the extent of adsorption [58]. However, β-CD-NKBC-1.5 still demonstrated a high removal rate for TC and MB and good preferential adsorption capacity for TC and MB when Cu^2+^ and Cr^6+^ were present (Figure 11d). This makes it suitable for TC or MB wastewater containing multiple coexisting ions. Figure 11c,d show the adsorption performance of β-CD-NKBC-1.5 for various contaminants, including dyes (MO and RhB), antibiotics (DOX and CIP), and heavy metals (Cu^2+^ and Cr^6+^).

### 2.7. Recycling Performance of β-CD-NKBC-1.5

**Figure 12:**
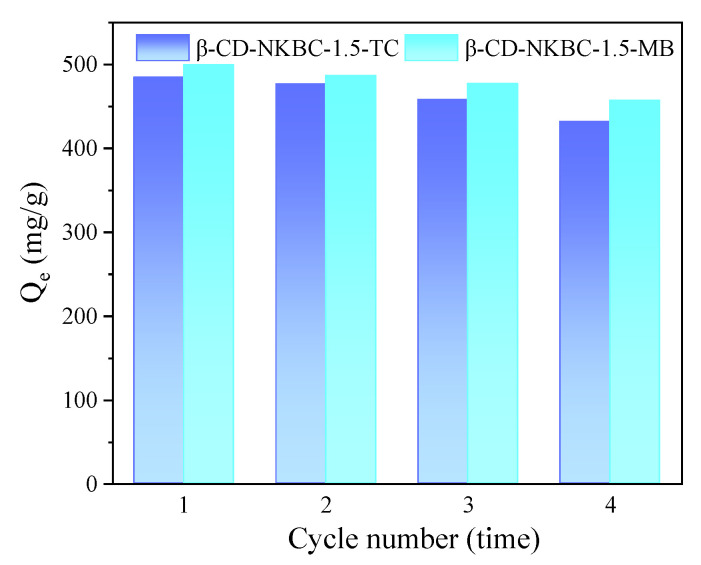
Adsorption–desorption cycle of β-CD-NKBC-1.5.

Absorbent recycling is an important performance measure in practical applications. Figure 12 displays the regeneration performance of β-CD-NKBC-1.5. The results revealed that the adsorption efficiency of β-CD-NKBC-1.5 could reach 96.94%, 95.30%, 91.62%, and 86.37% for TC, and 99.96%, 97.29%, 95.46%, and 91.47% for MB after the first to four cycles, respectively. With four cycles, β-CD-NKBC-1.5 adsorption on TC/MB reached 431.9/457.3 mg/g, which was 89.10%/91.50% of the first cycle, indicating strong reusability and stability. The removal efficiency gradually decreased with increasing cycle number, which could be attributed to pore blockage, occupation of active sites, and partial dissolution of the functional modifier during repeated use. The results indicate that β-CD-NKBC-1.5 has high reusability for practical applications.

### 2.8. Adsorption Mechanism of β-CD-NKBC-1.5

**Figure 13:**
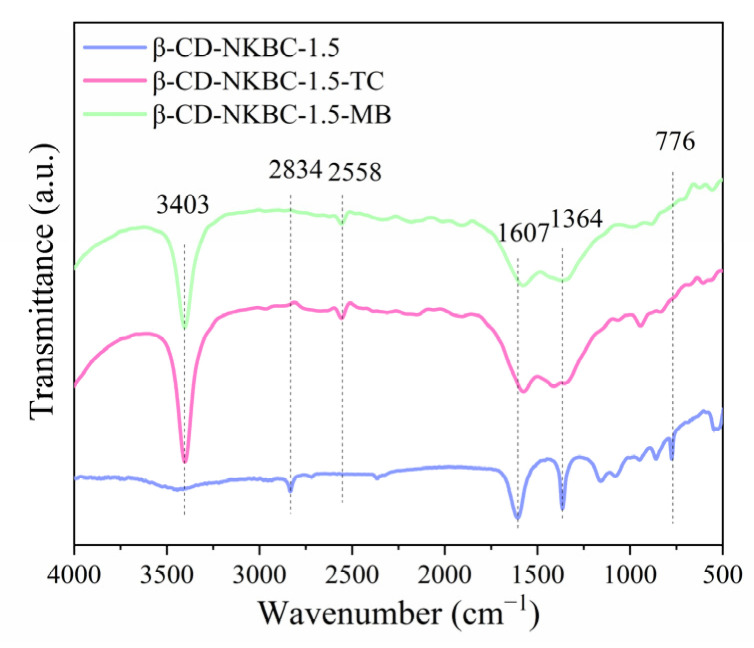
FTIR spectra of β-CD-NKBC-1.5 pre- and post-adsorption.

**Figure 14:**
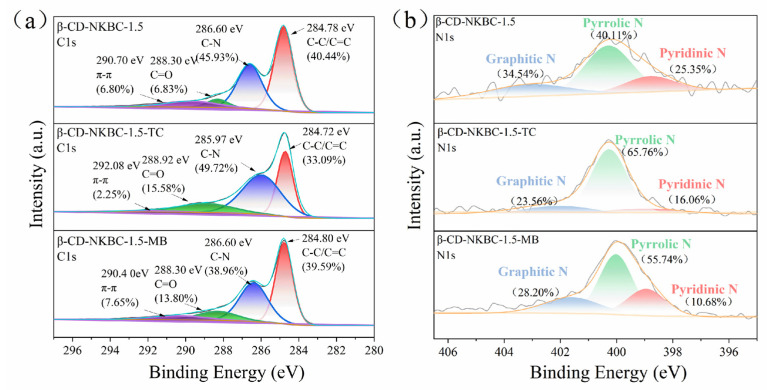
XPS of β-CD-NKBC-1.5 pre- and post-adsorption: (**a**) C1s, (**b**) N1s.

**Figure 15:**
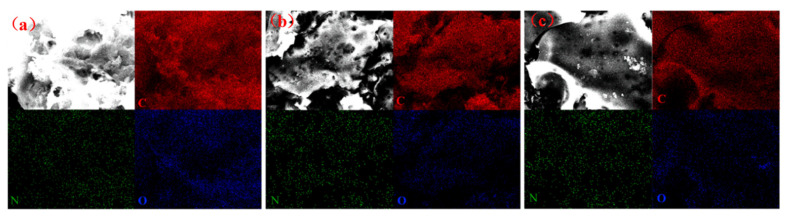
Mapping of β-CD-NKBC-1.5 before and after adsorption: (**a**) β-CD-NKBC-1.5; (**b**) β-CD-NKBC-1.5-TC; (**c**) β-CD-NKBC-1.5-MB.

After TC and MB adsorption, the β-CD-NKBC-1.5 composite was characterized by BET, FTIR, XPS, and elemental mapping (Figure 13, Figure 14 and Figure 15). The surface area, pore size distribution, and pore volume of β-CD-NKBC-1.5 changed after TC and MB adsorption. As indicated in Table 2, upon the adsorption of TC and MB, the specific surface area experienced a notable decrease, dropping from 1943 m^2^/g to 1440 m^2^/g and then to 1117 m^2^/g. Similarly, the pore volume saw a significant reduction, shrinking from 0.997 cm^3^/g to 0.869 cm^3^/g and further to 0.611 cm^3^/g. This data implies that pore filling is a pivotal factor in this transformation.

Figure 13 shows FTIR spectra for pre- and post-adsorption of TC and MB. The increased vibrational peak strength at 3403 cm^−1^ is due to the enrichment of −OH on the surface of β-CD and biochar, the introduction of −NH_2_ through N doping, and the presence of polar groups in TC and MB molecules. Hydrogen bonding between –OH groups and the –NH_2_ of TC/MB enhances the dipole moment of O–H/N–H bonds, leading to an increased peak intensity at 3403 cm^−1^. This highlights the importance of hydrogen bonds in the adsorption process. The peak at 1607 cm^−1^ weakens and shifts to a lower wavenumber due to π–π interactions between the aromatic rings of TC/MB and the carbon framework of β-CD-NKBC-1.5. The weakening of the absorption peak at 1364 cm^−1^ is due to the positively charged MB, which combines with the negatively charged N-containing groups (e.g., pyridinic N) on the surface of the material by electrostatic attraction [59]. The weakening of the absorption peak at 1364 cm^−1^ is due to the positively charged MB, which combines with the negatively charged N-containing groups (e.g., pyridinic N) on the surface of the material by electrostatic attraction. This electrostatic effect changes the chemical environment around the N-containing groups, weakening the vibration of the C–N bonds associated with them [60], leading to a decrease in the intensity of the absorption peak at 1364 cm^−1^. In the case of TC, its binding to the C–N group via hydrogen bonding or coordination (certain atoms in the TC molecule form coordination bonds with the N atom in the C–N group) changes the vibrational properties of the C–N bond, leading to a weakening of the absorption peak.

Figure 14 shows that, after adsorption with TC and MB, the fraction of C–C bonds in β-CD-NKBC-1.5 fell from 48.58% to 33.09% and 47.67%, respectively. This indicates that π–π interactions between the aromatic rings of TC/MB and the carbon skeleton involve some C–C bonds in the conjugation system, reducing their proportion and confirming the key role of π–π stacking. Furthermore, the percentage of π–π increased after MB adsorption. The π–π peak is associated with electronic transitions in conjugated systems. The increase in the percentage indicates that a more extensive π–π stacking structure was formed between the material and MB molecules, and the electrons were more active in the conjugated system, which further proves the importance of π–π stacking in MB adsorption.

The adsorption of TC and MB by β-CD-NKBC-1.5 resulted in a drop in the percentage from 23.53% to 16.06% and 10.68%, respectively, indicating that pyridinic N plays a role in the adsorption process. Pyridinic N has a lone pair of electrons and can act as a Lewis base site for interaction with pollutant molecules [61]. Meanwhile, the proportion of pyridinic N increased dramatically, from 40.11% to 65.76% to 55.74%, indicating that some pyridinic N was transformed into pyrrolic N during adsorption. This transition could be caused by an alteration in the chemical environment around the N atom following the interaction of the contaminant molecule with pyridinic N. β-CD-NKBC-1.5 improved TC and MB capture through Lewis acid–base mechanisms. The graphite N concentration fell from 34.54% to 23.56% (after TC adsorption) and 28.20% (after MB adsorption). Graphitic N, with its high electronegativity, acts as a π-electron acceptor and interacts strongly with the aromatic rings (π–donors) of TC and MB, leading to its involvement in the conjugated system and changes in its chemical environment.

Figure 15 clearly indicates a rise in the nitrogen elemental concentration within β-CD-NKBC-1.5 following the adsorption of both TC and MB, a phenomenon that can be attributed to the microporous and mesoporous structure of the material. The N–containing groups (e.g., amino group of TC, sulfur–azide heterocycle of MB) of TC/MB molecules enter into the pore space during adsorption, and their N–doped sites (e.g., pyridinium nitrogen, and the amino group) in the inner wall of the pore space undergo a localized enrichment. The adsorption mechanism of β-CD-NKBC-1.5 on TC and MB is a complex multifactorial and synergistic process, including the physical effects of pore filling, as well as a variety of chemical effects such as hydrogen bonding, π–π conjugation, electrostatic interactions, and Lewis acid–base reactions.

To quantitatively evaluate the contribution of key surface functional groups to the adsorption of TC and MB, the relative contents of C– and N–containing species derived from XPS analysis before and after adsorption were correlated with the adsorption capacity. Given that the total adsorption capacity arises from the combined effect of multiple functional groups, a contribution analysis was performed based on the variation in each group’s content (*Δ*, in at%) and its relative chemical activity in pollutant binding. The contribution ratio (${CR}_{i}$) for each functional group was calculated using Equation (1):

(1) $${CR}_{i}=\frac{W_{i}\cdot \left|{\Delta X}_{i}\right|}{\sum_{j}W_{j}\cdot \left|{\Delta X}_{j}\right|}\times 100\%$$

where $W_{i}$ is the weight factor assigned according to the interaction strength (e.g., Lewis acid–base, π–π stacking, and hydrogen bonding) reported in the literature and this study, and ${\Delta X}_{i}$ is the change in the atomic percentage of group i before and after adsorption.

As summarized in Table 7, for TC adsorption, pyridinic N and π–π interactions contributed the most, with contribution rates of 29.8% and 29.7%, respectively, followed by graphitic N (17.0%). For MB adsorption, π–π stacking (31.6%) and pyridinic N (29.4%) also played dominant roles, while C–N species contributed 14.5%. The results confirm that chemisorption mechanisms, particularly π–π interactions and Lewis acid–base interactions, govern the adsorption process, consistent with the kinetic and isotherm analyses. The minor contribution from C–C and C=O groups suggests that pore filling and hydrogen bonding serve as supplementary pathways.

## 3. Materials and Methods

### 3.1. Materials and Reagents

Cotton stalks were sourced from Kashgar farms in Xinjiang, and their composition is detailed in Table 8. Shanghai, China, Chemical Technology Co., Ltd. supplied β-cyclodextrin (C_42_H_70_O_35_, 1134.98 g/mol) with a purity of ≥99.7%. Tianjin Fuchen, China, Chemical Reagents Co., Ltd. supplied epichlorohydrin (ECH, C_3_H_5_ClO, 92.52 g/mol), AR grade. Deionized water was produced in house by the laboratory. TC (C_22_H_24_N_2_O_8_, 444.45 g/mol), MB (C_16_H_18_N_3_ClS·3H_2_O,373.90 g/mol), KOH, and melamine monomer were supplied by Adamas-beta, AR grade. HCl, NaOH, MgCl_2_, KCl, Na_2_SO_4_, CaCl_2_, NaH_2_PO_4_, and Na_2_CO_3_ were all purchased from Shanghai, China, Greagent Company, with AR grade purity. Anhydrous ethanol was supplied by Shanghai, China, Greagent Ltd., with a purity of ≥99.95%. N_2_ was supplied by Xinjiang, China, Urumqi Jinhongshan Company, with a purity of ≥99.99%.

### 3.2. Preparation of Biochar

Melamine is an effective source for doping carbon materials because of its high N content (about 66% by mass); it has a triazine ring structure that undergoes stepwise decomposition, forming intermediates such as melam, melem, and melon, thereby improving the N doping efficiency in the carbon material. The melamine monomer, KOH, and cotton stalks were mixed at a mass ratio of 5:1:1 in a corundum boat and ground thoroughly. The mixture was subsequently heated in a tube furnace at a ramp rate of 5 °C/min until reaching 900 °C, where it underwent pyrolysis for two hours while being continuously purged with nitrogen gas. Biochar was treated with 0.1 mol/L HCl at 80 °C, washed with pure water, and then dried in an oven at 105 °C for 12 h to produce NKBC. Then, 5 g of β-CD and 2.4 g of epichlorohydrin (ECH) were dissolved in 50 mL of 7% (*w*/*v*) NaOH solution and stirred at 120 rpm for 6 h at room temperature. Following this procedure, we incorporated 2.0, 1.5, and 1.0 g of NKBC into the solution. The mixture was gently stirred at ambient temperature for 5 h and then left to sit undisturbed for 24 h. The resulting product was rinsed with distilled water and dried at 70 °C for 24 h. The newly formed compounds were thereafter identified as β-CD-NKBC-2, 1.5, and 1, respectively.

### 3.3. Characterization

β-CD-NKBC/NKBC were characterized via SEM, FTIR, Raman, BET, and XPS analyses. The microstructure of the sample was examined using a field-emission scanning electron microscope (SEM, Hitachi S4800, Tokyo, Japan) at a magnification of 2000×. Biochar functional groups were examined using Fourier transform infrared spectroscopy (FTIR, Great EMB Model 10 from Tianjin, China) with the KBr press method in the range of 500–4000 cm^−1^. The β-CD-NKBC element’s valence was determined via ESCALAB 250Xi XPS (Thermo Fisher Scientific, Waltham, MA, USA). N_2_ adsorption–desorption isotherms were measured using an ASAP 2460 analyzer (Micromeritics, Norcoss, GA, USA) at 77 K after degassing at 473.15 K. A confocal Raman spectrometer (Raman, HORIBA, Montpellier, France) was used to examine the flaws and graphitization of biochar at a wavelength of 532 nm and in a spectrum range of 800–2000 cm^−1^.

### 3.4. Experimental Section

#### 3.4.1. Adsorption Performance Experiment

We added 5 mg of β-CD-NKBC (or NKBC) to 50 mL of TC or MB solution (50 mg/L) at a controlled temperature in the dark. The supernatant was filtered, and the residual TC or MB concentration was measured using a UV–Vis spectrophotometer at 356 nm or 664 nm, respectively. Equations (S1) and (S2) were used to quantify the adsorption and removal rates, respectively (Supplementary Materials).

#### 3.4.2. Effect of pH on Adsorption Performance

We measured 5 mg of β-CD-NKBC (or NKBC) and introduced it into a TC or MB solution (50 mg/L) at different pH levels; the experiment was carried out by following the protocol outlined in Section 3.4.1.

#### 3.4.3. Adsorption Kinetics

We added 10 mg of β-CD-NKBC (or NKBC) to 100 mL of 50 mg/L TC or MB solution at the optimal pH. The solution was then placed in a temperature-controlled oscillator and spun at 180 revolutions per minute in the dark. At predetermined time intervals (1, 5, 10, 15, 20, 30, 60, 90, 120, 180, 240, 360, 540, 720, and 1440 min), 5 mL of supernatant was collected using a pipette and filtered through a 0.45 μm PTFE membrane for analysis. The filtrate was then tested using a UV–visible spectrophotometer to determine the concentration at each time point. The adsorption kinetics were analyzed using pseudo-first-order, pseudo-second-order, and Weber–Morris intraparticle diffusion models. The equations derived from fitting are depicted in Equations (S3)–(S5) (Supplementary Materials).

#### 3.4.4. Adsorption Isotherm

We added 5 mg of β-CD-NKBC to 50 mL of TC solution (20, 40, 60, 80, 100, 140, and 200 mg/L) or MB solution (30, 40, 50, 60, 70, 80, and 100 mg/L). Adsorption was carried out under light-avoiding conditions, oscillating at 25 °C, for 24 h. Three different isotherm models were used in this study: Langmuir, Freundlich, and Dubinin–Radushkevich, and the fitted equations are presented in Equations (S6)–(S11) (Supplementary Materials).

#### 3.4.5. Adsorption Thermodynamics Experiment

We combined 5 mg of β-CD-NKBC-1.5 with 50 mL of TC solution (20–200 mg/L)/MB solution (30–100 mg/L) at various concentrations. The solution was shaken for 24 h in a constant-temperature shaker at temperatures of 288.15 K, 298.15 K, and 308.15 K. Thermodynamic parameters were calculated to understand the spontaneity and nature of the adsorption process. The van’t Hoff equation was employed, as shown in Equations (S12)–(S14) (Supplementary Materials).

#### 3.4.6. Regenerative Adsorption Experiment

We added 10 mg of β-CD-NKBC-1.5 to 100 mL of 50 mg/L TC or MB solution and shook it for 24 h to reach adsorption equilibrium. The absorbance of the supernatant was measured, β-CD-NKBC-1.5 was recovered, and TC was desorbed with 0.2 mol/L NaOH solution for 24 h (anhydrous ethanol desorbed MB). The adsorbent was separated by filtration, washed, and dried. The experimental approach was repeated four times to determine the regeneration performance of β-CD-NKBC-1.5.

#### 3.4.7. Different Conditions Adsorption Performance Experiments

Solutions containing various cations and anions at concentrations of 100–500 mg/L were prepared at the optimal pH. β-CD-NKBC was incubated in the salt solutions (NaH_2_PO_4_, NaCl, Na_2_CO_3_, Na_2_SO_4_, KCl, CaCl_2_, and MgCl_2_) in a constant-temperature shaker for 24 h. The supernatant was then collected for absorbance measurement.

## 4. Conclusions

In this study, a novel β-cyclodextrin-functionalized nitrogen-doped biochar was successfully synthesized via molten salt activation combined with surface grafting modification. The incorporation of β-CD significantly optimized the pore structure and surface chemical properties of the biochar, yielding a high specific surface area of 1943 m^2^/g and abundant nitrogen-containing functional groups. The as-prepared β-CD-NKBC-1.5 composite exhibited outstanding adsorption performance towards TC and MB, with maximum adsorption capacities of 1269.8 mg/g and 969.4 mg/g at 308.15 K, respectively—substantially outperforming most previously reported biochar-based adsorbents.

Kinetic studies revealed that the adsorption processes followed the pseudo-second-order model, suggesting that chemisorption governs the rate-limiting step. Isotherm fitting indicated that the Langmuir model best described the adsorption behavior, implying monolayer coverage on a homogeneous surface. Thermodynamic analysis confirmed that the adsorption of both TC and MB was spontaneous and endothermic in nature. Comprehensive characterization using BET, FTIR, XPS, and elemental mapping before and after adsorption revealed that the removal mechanisms involved multiple synergistic interactions, including pore filling, hydrogen bonding, π–π stacking, electrostatic attraction, and Lewis acid–base interactions. Notably, pyridinic N and graphitic N played critical roles as active sites, facilitating electron donor–acceptor interactions with the aromatic structures of TC and MB.

Furthermore, β-CD-NKBC-1.5 demonstrated excellent regenerability, maintaining over 86% and 91% of its initial adsorption capacity for TC and MB after four consecutive cycles, respectively. The material also exhibited stable adsorption performance across a wide pH range (2–10) and in the presence of competing ions (Na^+^, K^+^, Ca^2+^, Mg^2+^, SO_4_^2−^, etc.) at concentrations up to 500 mg/L, highlighting its potential for application in complex wastewater matrices.

In summary, this work presents a promising strategy for the functionalization of agricultural waste-derived biochar through the synergistic combination of molten salt activation, nitrogen doping, and β-CD grafting. The resulting composite not only achieves superior adsorption capacity for both antibiotic and dye pollutants but also offers excellent stability and reusability, positioning it as a sustainable and efficient adsorbent for advanced water purification.

## Figures and Tables

**Table 1:** Surface area analysis of NKBC and β-CD-NKBC.

Sample	Specific Surface Area (m^2^/g)	Microporous Specific Surface Area (m^2^/g)	Total Pore Volume (cm^3^/g)	Microporous Pore Volume (cm^3^/g)	Average Pore Size (nm)	Average Mesopore Diameter (nm)
NKBC	3156	2967	1.583	1.367	2.006	2.672
β-CD-NKBC-2	1773	1630	0.957	0.805	2.159	2.741
β-CD-NKBC-1.5	1943	1825	0.997	0.866	2.053	2.708
β-CD-NKBC-1	711	610	0.507	0.396	2.852	3.289
β-CD-NKBC-1.5-TC	1440	1284	0.869	0.633	2.287	3.153
β-CD-NKBC-1.5-MB	1117	1002	0.611	0.497	2.187	3.059

**Table 2:** Comparison of adsorption capacities for TC and MB across different biochar materials.

Carbon Source	Modification Methods	Materials	Emerging Contaminants	Adsorption Capacity (mg·g^−1^)	Ref.
Polyurethane prepolymer	Cross-linked carboxymethyl chitosan	WPU-CMCS10	MB	222.65	[42]
Acorn shells	500 °C, Na_2_CO_3_	QCAC	MB	279.82	[43]
Sugarcane bagasse	Melamine nitrogen doping	NPC2	MB	581.40	[21]
Papermaking sludge	High-temperature pyrolysis	PSBCs	TC	76.39	[44]
Cotton stalks	KOH activation	KBCl-900	MB/TC	912.21/845.00	[10]
Corncob	Melamine nitrogen doping	Fe1N2KBC	TC	764.35	[45]
Poplar wood powder	1-methyl-3-methylimidazolium tetrachloromanganate-modified biochar	Mn/N-BC	TC	201.50	[46]
Rape straw	Pyrolysis at 800 °C	GBC800	TC	16.97	[47]
	Potassium bicarbonate activation	KGBC800		294.86	
	potassium bicarbonate and urea activation	N-KGBC800		604.71	
Bamboo powder	PVC modification	IFPHC	MB	613.94	[48]
Temple flower	KOH activation	Fe_3_O_4_@TEB	MB	113.35	[49]
Walnut shells	NH_4_H_2_PO_4_ modification	NPC-1	MB	263.30	[50]
Pine Wood	Phosphoric acid modification	LPHC	TC	120.63	[51]
Corn straw	β-cyclodextrin modification	β@MHBC	TC	67.18	[52]
Carbonation of tris(4-formylphenyl)amine onto β-cyclodextrin		TFPA-N-β-CD	MB/TC	272.03/33.89	[53]
Cotton stalks	β-CD-modified N-doped biochar	β-CD-NKBC-1.5	MB/TC	969/1269	This work

**Table 3:** Parameters of pseudo-first-order and pseudo-second-order model fits of β-CD-NKBC-1.5 to TC and MB.

Samples	Q_e_, Exp (mg/g)	Pseudo-First-Order Model			Pseudo-Second-Order Model		
		K_1_ (min^−1^)	Q_e_, cal (mg/g)	R^2^	K_2_ (min^−1^)	Q_e_, cal (mg/g)	R^2^
β-CD-NKBC-1.5-TC	497.421	0.039	477.805	0.962	1.080 × 10^−4^	497.146	0.997
β-CD-NKBC-1.5-MB	499.671	0.050	491.669	0.866	1.869 × 10^−4^	499.273	0.992

**Table 4:** Parameters for fitting the internal diffusion model for β-CD-NKBC-1.5.

Samples	K_int1_ (mg/(g·min^0.5^))	C_1_	R^2^	K_int2_ (mg/(g·min^0.5^))	C_2_	R^2^	K_int3_ (mg/(g·min^0.5^))	C_3_	R^2^
β-CD-NKBC-1.5-TC	47.630	34.588	0.992	11.299	327.949	0.980	0.511	479.948	0.789
β-CD-NKBC-1.5-MB	41.133	106.990	0.974	9.713	354.811	0.966	0.653	482.739	0.913

**Table 5:** Parameters of Langmuir, Freundlich, and D-R model fits of β-CD-NKBC-1.5 to TC and MB.

Samples	T (K)	Langmuir			Freundlich			Dubinin–Radushkevich	
		Q_m_ (mg/g)	K_L_ (L/mg)	R^2^	K_F_ (mg/g)	n_F_	R^2^	E (KJ/ mol)	R^2^
β-CD-NKBC-1.5-TC	288.15	1165.7	0.645	0.991	495.2	4.807	0.807	1.426	0.894
	298.15	1232.8	0.933	0.994	636.6	5.175	0.881	1.663	0.881
	308.15	1269.8	1.392	0.993	840.2	6.523	0.911	5.617	0.980
β-CD-NKBC-1.5-MB	288.15	915.2	4.395	0.995	702.1	3.851	0.897	8.716	0.950
	298.15	961.5	239.346	0.993	960.7	6.950	0.945	10.588	0.964
	308.15	969.4	418.377	0.997	1212.6	5.530	0.987	11.279	0.971

**Table 6:** Thermodynamic constants of β-CD-NKBC-1.5 for TC and MB.

Samples	ΔG^0^ (KJ/mol)			Enthalpy Change	Entropy Change
	288.15 K	298.15 K	308.15 K	ΔH^0^ (KJ/mol)	ΔS^0^ (J/(mol·K))
β-CD-NKBC-1.5-TC	−8.494	−9.326	−9.974	12.850	74.171
β-CD-NKBC-1.5-MB	−15.232	−18.906	−22.721	92.650	374.152

**Table 7:** Quantitative contribution of surface functional groups to TC and MB adsorption on β-CD-NKBC-1.5.

Functional Group	Weight Factor	ΔTC (%)	Contribution to TC (%)	ΔMB (%)	Contribution to MB (%)
Pyridinic N	1.0	25.65	29.8	15.63	29.4
Graphitic N	1.0	−14.67	17.0	9.29	17.5
π–π	1.0	−25.61	29.7	16.83	31.6
Pyrrolic N	0.6	10.98	7.6	−6.34	7.1
C–N	0.6	−7.47	5.2	12.85	14.5
C=O	0.2	−2.64	1.5	−3.05	2.90
C–C	0.2	−15.49	3.6	−0.91	0.3
Total			94.4		103.30 *

* Sum slightly exceeds 100% due to weight factor normalization; values represent relative importance.

**Table 8:** Content of major elements in cotton stalks.

Sample	C (%)	H (%)	O (%)	N (%)	S (%)	Fe (‰)	Si (‰)
Cotton stalks	44.85	5.73	44.74	0.95	0.37	0.44	1.56

## Data Availability

The original contributions presented in this study are included in the article/Supplementary Materials. Further inquiries can be directed to the corresponding authors.

## Supplementary Materials

The following supporting information can be downloaded at: https://www.mdpi.com/article/10.3390/molecules31081284/s1. Equations (S1) and (S2): Adsorption performance; Equations (S3)–(S5): Adsorption kinetics equation; Equations (S6) and (S7): Langmuir modal; Equation (S8): Freundlich model; Equations (S9)–(S11): Dubinin–Radushkevich model; Equations (S12)–(S14): van’t Hoff equation.
