# Supplementary material for: Study on β-Cyclodextrin-Functionalized Molten Salt Nitrogen-Doped Biochar and Its Adsorption Performance and Mechanism
Source: Molecules. 2026 Apr 14;31(8):1284. doi: 10.3390/molecules31081284 (PMC13119241; doi:10.3390/molecules31081284)
Supplement: Supplementary file 1 [file molecules-31-01284-s001.zip › molecules-4186744-supplementary.pdf]

Supporting Information

Study on β-Cyclodextrin-Functionalized Molten Salt Nitrogen-Doped Biochar and Its Adsorption Performance and Mechanism

Sining Li[1, †], Yong Huang[1, 3, † *], Qiushuang Cui[1, 2, †], Ke Jin[4], Hanyu Wei[1], Wen Liu[1], Huan Li[1], and , Ruyun Bai[1]

1 State Key Laboratory of Chemistry and Utilization of Carbon Based Energy Resources, College of Chemistry, Xinjiang University, Urumqi, 830017, Xinjiang, PR China

2 Fangda Special Steel Technology Co., Ltd., Nanchang, 330012, jiangxi, PR China

3 College of Civil Engineering and Architecture, Xinjiang University, Urumqi, 830017, Xinjiang, PR China

4 Henan Province Water Conservancy Second Engineering Bureau Group Co., Ltd, Zhengzhou, 450016, Henan, PR China

† These authors contributed equally to this work.

Corresponding author: pengyou0991@163.com

1. Adsorption performance

$$q_t=\frac{(C_0-C_t)\times V}{m} \quad (S1)$$

$$\text{Removal efficiency} =\frac{C_0-C_t}{C_0} \quad (S2)$$

where qt and Ct represent the adsorbed amount and TC/MB concentration at the prescribed time (t); C_0, V, and m represent the initial concentration, volume, and adsorbent of TC (MB) in the reaction system, respectively.

2. Adsorption kinetics:

2.1 Pseudo-first-order:

$$\ln(Q_e - Q_t) = \ln Q_e - K_1 t \quad (S3)$$

Where K_1 (min^{-1}) was the pseudo-first order adsorption rate constant.

2.2 Pseudo-second order:

$$t/Q_t = 1/K_2 {Q_e}^2 + t/Q_e$$

(S4)

Where K_2 (g/(mg·min)) was the pseudo-second order adsorption rate constant.

2.3 Webber-Morris:

$$Q_t = K_i t^{1/2} + C_i \quad (S5)$$

Where K_i (mg/(g·$min^{0.5}$)) was the adsorption rate constants of intra-particle diffusion model and C_i was the constant for film thickness of the intra-particle diffusion model.

3. Adsorption isotherms:

3.1 Langmuir modal:

$$Qe = \frac{q_{max} K_L C_e}{1+K_L C_e} \quad (S6)$$

Where K_L (L/mg) was the Langmuir constant, q_{max} (mg/g) was the maximum adsorption capacity.

The separation factor (R_L) for Langmuir model is used to evaluate the adsorption process, It showed that the isotherm was unfavorable (R_L>1), favorable (R_L<1), linear (R_L=1), or irreversible (R_L=0), which is defined as follow:

$$R_L = \frac{1}{1+K_L C_m} \quad (S7)$$

Where C_m was the initial concentration.

3.2 Freundlich model:

$$Q_e = K_F C_e^{1/n} \quad (S8)$$

Where K_F and 1/n was the Freundlich constants.

3.3 Dubinin–Radushkevich model:

$$\ln Q_{eD} = \ln Q_D - B_D \varepsilon^2 \quad (S9)$$

$$\varepsilon = RT\ln(1+\frac{1}{C_{eD}}) \quad (S10)$$

Where Q_{eD} was the maximum adsorption capacity (mmol/g), and BD was Dubinin–Radushkevich isotherm constant (mol^2/kJ^2), R represented ideal gas constant (8.314 J/(mol·K)), T mean temperature (K), and C_{eD} represented equilibrium solution concentration (mmol/L).

The free adsorption energy (BD) was further used for the calculation of the mean adsorption energy (E, kJ/mol) to predict the occurrence of the ion-exchange mechanism by the following equation:

$$E = \frac{1}{\sqrt{2B_D}} \quad (S11)$$

The magnitude of 1 <E < 8 kJ/mol showed that the adsorption was mainly physical process, and the magnitude of 8 < E < 16 kJ/mol was mainly chemical adsorption.

3.4 van't Hoff equation:

$$K_c = \frac{Q_e}{C_e} \quad (S12)$$

$$\Delta G^0 = -RT \times \ln K_c \quad (S13)$$

$$\ln K_c = \frac{\Delta S^0}{R} - \frac{\Delta H^0}{RT} \quad (S14)$$

Where K_c is the dispersion coefficient of the adsorption process, ΔS^0 (J/mol/K), ΔH^0 (KJ/mol) and ΔG^0 (KJ/mol) are the entropy, enthalpy and the standard Gibbs free energy change, respectively, and R (8.314 J/mol/K) is the universal gas constant.
